# Luteolin sensitizes the antitumor effect of cisplatin in drug-resistant ovarian cancer via induction of apoptosis and inhibition of cell migration and invasion

**DOI:** 10.1186/s13048-018-0468-y

**Published:** 2018-11-19

**Authors:** Haixia Wang, Youjun Luo, Tiankui Qiao, Zhaoxia Wu, Zhonghua Huang

**Affiliations:** 1 Department of Obstetrics and Gynecology, Jinshan branch of Shanghai Sixth People’s Hospital, Shanghai Jiaotong University, Shanghai, China; 2 Department of Oncology, Jinshan Hospital, Fudan University, Shanghai, China; 3 Department of Traditional Medicine, Jinshan branch of Shanghai Sixth People’s Hospital, Shanghai Jiaotong University, Shanghai, China

**Keywords:** Luteolin, Cisplatin-resistant ovarian cancer, Apoptosis, Migration, Invasion

## Abstract

Luteolin, a polyphenolic flavone, has been demonstrated to exert anti-tumor activity in various cancer types. Cisplatin drug resistance is a major obstacle in the management of ovarian cancer. In the present study, we investigated the chemo-sensitizing effect of luteolin in both cisplatin-resistant ovarian cancer cell line and a mice xenotransplant model. In vitro, CCK-8 assay showed that luteolin inhibited cell proliferation in a dose-dependent manner, and luteolin enhanced anti-proliferation effect of cisplatin on cisplatin-resistant ovarian cancer CAOV3/DDP cells. Flow cytometry revealed that luteolin enhanced cell apoptosis in combination with cisplatin. Western blotting and qRT-PCR assay revealed that luteolin increased cisplatin-induced downregulation of Bcl-2 expression. In addition, wound-healing assay and Matrigel invasion assay showed that luteolin and cisplatin synergistically inhibited migration and invasion of CAOV3/DDP cells. Moreover, in vivo, luteolin enhanced cisplatin-induced reduction of tumor growth as well as induction of apoptosis. We suggest that luteolin in combination with cisplatin could potentially be used as a new regimen for the treatment of ovarian cancer.

## Introduction

Ovarian cancer is one of the most common malignant tumors of gynecology, with the highest mortality compared with other gynecologic cancer because of its acute onset, rapid progress and high metastasis rate [1, 2]. Epithelial ovarian cancer (EOC) accounts for 85–90% of total ovarian carcinoma and is the most aggressive one. In early stage, surgical resection combined with chemotherapy is an effective therapy method [3]. Unfortunately, most of the patients reach advanced stage at the time of diagnosis [4, 5]. For patients with advanced EOC, platinum-based chemotherapy is the standard of care. More than 80% of ovarian tumors response to first-line platinum-based therapy [6], however, the majority of patients acquire resistance to cisplatin (CDDP) treatment and ultimately result in relapse and poor prognosis [7, 8]. Therefore, it is necessary to develop appropriate combined reagents to solve drug resistance and enhance the sensitivity of EOC to cisplatin treatment.

Chemotherapy resistance is a key factor that limits the cure rate of ovarian cancer. The mechanisms underlying cancer cells resistance to cisplatin are not fully understood. It is acknowledged that various mechanisms are responsible for drug-resistance, including the decrease of the effective concentration of drugs in cells, the abnormalities of drug targets, and the abnormal regulation of cell apoptosis [9]. Currently, there are some ways to overcome the chemo-resistance, such as maintenance therapy, novel cytotoxic agents, modulation of apoptosis and combination therapy [10]. Natural medicine, with its small side effects and significant therapeutic effect, attracts a lot attention as a potential combination agent for cisplatin treatment.

Luteolin is one of the most common flavonoid compound that is widely existed in various plants including peppermint, rosemary, thyme, pinophyte, and pteridophyta [11]. Numerous studies suggested that luteolin possesses a variety of pharmacological properties including anti-inflammatory, antiallergic, antioxidant, antimicrobial, immune regulation and anticancer activities [11, 12]. Among all these properties, anti-tumor effect has attracted a lot of attention. Researchers have found that luteolin exerts anti-tumor activities via several mechanisms, including cell cycle arrest, apoptosis induction, angiogenesis and metastasis inhibition [13–16]. A previous study has demonstrated that luteolin can sensitize oxaliplatin-resistant colorectal cancer cells to chemotherapeutic drugs through the inhibition of the Nrf2 pathway [17]. Another study reported that luteolin can be used as a chemosensitizer to improve the therapeutic effect of tamoxifen in drug-resistant human breast cancer cells via the inhibition of cyclin E2 expression [18]. These results suggest that luteolin exhibits potential chemosensitivity property for various cancers. However, whether luteolin can increase the chemotherapy sensitivity of cisplatin-resistant ovarian cancer and the underlying mechanisms is rarely reported, which needs to be further studied.

In the current study, we investigated the synergistic effects of luteolin combined with cisplatin in drug-resistant ovarian cancer cell line CAOV3/DDP both in vitro and in vivo, and tried to explore associated molecular mechanisms.

## Materials and methods

### Reagents and cell lines

Luteolin was bought from Jin Sui Biological Technology (Shanghai, China). It was dissolved in DMSO as a stock of 500 mM and stored at − 20 °C. Cisplatin was purchased from QILU Pharmaceutical (Shandong, China). Human drug-resistant ovarian cancer cell line, CAOV3/DDP were obtained from the Shanghai Sixin Biotechnology company (Shanghai, China) and maintained in RPMI1640 (Gibco, Grand Island, NY, USA) containing 10% fetal bovine serum (Gibco, Grand Island, NY, USA). The cells were incubated at 37 °C in a humidified atmosphere with 5% CO_2_.

### Cell proliferation assay

Cell proliferation was measured using Cell Counting Kit-8 (CCK-8; Dojindo Molecular Technologies, Inc., Kumamoto,Japan). Briefly, CAOV3/DDP cells (5 × 10^3^) were seeded into 96-well plates and allowed for adhesion overnight. Then the cells were administrated with eight treatments as follows: control (culture medium); low-dose of luteolin (10 μM); medial-dose of luteolin (50 μM); high-dose of luteolin (100 μM); CDDP (2 μg/ml); CDDP (2 μg/ml) + low-dose of luteolin (10 μM); CDDP (2 μg/ml) + medial-dose of luteolin (50 μM); CDDP (2 μg/ml) + high-dose of luteolin (100 μM). After 48 h treatment, the culture medium was removed and CCK-8 was added according to the manufacturer’s instruction. Then the cells were incubated for 1–4 h at 37 °C and the absorbance was detected at 450 nm using a microplate reader. Cell proliferation was calculated as follows:

Cell proliferation (%) = [(OD of experiment group – OD of blank) / (OD of control group – OD of blank)] × 100%.

### Apoptosis analysis

Cell apoptosis was detected using Annexin V-FITC Apoptosis Detection Kit (BD Pharmingen, Franklin Lakes, NJ, USA). Cells (2 × 10^4^) were seeded into 6-well plates and treated with various concentration of luteolin (0, 10, 50, 100 μM) or CDDP alone or in combination for 48 h. Then both the adherent and floating cells were harvested and stained according to the manufacturer’s protocol. The apoptosis rate was analyzed by flow cytometry.

### Wound-healing assay

Cell migration ability was measured by wound-healing assay. Briefly, cells were seeded into 6-well plates and allowed to grow to a monolayer. Subsequently, a straight scratch was generated across the plate using a 200 μl pipet tip. The cells were washed with PBS and incubated with various concentration of luteolin (0, 10, 50, 100 μM) and CDDP alone or in combination (dissolve the chemicals in serum-free culture medium). Wound healing was observed and photographed at 0 and 48 h.

### Matrigel invasion assay

The Matrigel was diluted in serum-free RPMI-1640 (RPMI-1640: Matrigel = 8:1) and added into the upper chamber. After treatment with various concentrations of luteolin (0, 10, 50, 100 μM) and CDDP alone or in combination for 48 h, the cells (5 × 10^4^) were trypsinized and collected. 5 × 10^4^ cells in 200 μl serum-free medium were seeded into the upper chamber. The lower chamber was filled with 600 μl complete medium containing 10% FBS. After incubation for 48 h, the invaded cells were stained with crystal violet and pictured under a microscope at  x100 magnification.

### qRT-PCR

After treatment, the medium was removed and the cells were washed with PBS. The total RNA of each group was extracted using TRIzol (Invitrogen, California, USA). Then the RNA was reversely transcribed to cDNA using the PrimeScript™ RT Reagent kit (Takara, Dalian, China) according to the manufacturer’s instruction. The qPCR was performed using a SYBR Premix Ex Taq (Tli RNaseH Plus) in Applied Biosystem 7300 (Applied Biosystems, Foster city, CA, USA). The BCL-2 mRNA expression was analyzed using the 2-ΔΔCq method taking β-Actin as reference. The gene primer sequences were shown in Table 1.

**Table 1:** Primer sequences for genes

Gene	Primer Sequences
BCL-2	F: 5’-AACATCGCCCTGTGGATGAC-3’
	R: 5’-AGAGTCTTCAGAGACAGCCAGGAG-3’
β-Actin	F: 5’-CATTGCCGACAGGATGCAG-3’
	R: 5’-CTCGTCATACTCCTGCTTGCTG-3’

### Western blot

CAOV3/DDP cells were seeded into 6-well plates (2 × 10^5^/well),and treated with increasing doses of luteolin (0, 10, 50, 100 μM) or cisplatin (2 μg/ml) or both for 48 h. Then, the cells were harvested, and total proteins were extracted using cell lysis buffer (1 mM PMSF, 50 mM Tris (pH 8.1), 1% SDS, sodium pyrophosphate, β-glycerophosphate, sodium orthovanadate, sodium fluoride, EDTA, leupeptin and other inhibitors) (Beyotime Biotechnology, Shanghai, China. No. P0013G). The protein concentration was detected using BCA assay (Mai Bio Co., Ltd.). 20 μg proteins of each group were separated on SDS-PAGE, and then transferred onto PVDF membranes (Millipore Corp., Bedford, MA, USA). Membranes were blocked with 5% non-fat dry milk, and probed with primary antibodies against Bax (1:4000, Cell Signaling Technology, USA), Bcl-2 (1:4000, Cell Signaling Technology, USA), and β-Actin (1:5000, ProteinTech Group, Inc., USA) at 4 °C overnight. Then the membrane was washed with PBS and incubated with HRP-conjugated secondary antibodies (1:5000) for 1 h at room temperature. Finally, the blots were imaged with ECL (EMD Millipore).

### In vivo xenograft experiment

Female BALB/c nude mice (5–6 weeks old) were obtained from the Shanghai Experimental Animal Center. Animals were raised in pathogen-free conditions at 22 °C, 50% humidity. Animal experiments were approved by the Institutional of Animal Care and Use Committee of Jinshan Hospital, Fudan University. The cisplatin resistant cell line CAOV3/DDP (5 × 10^6^ cells) in a volume of 100 μl of PBS were inoculated in the subcutaneous tissue of the nude mice. Two weeks after implantation, the tumors were visible and the mice were randomly allocated into 8 groups (6 mice per group): (1) control group (normal saline); (2) luteolin low-dose (10 mg·kg^− 1^·d^− 1^) group; (3) luteolin medial-dose (20 mg·kg^− 1^·d^− 1^) group; (4) luteolin high-dose (40 mg·kg^− 1^·d^− 1^) group; (5) CDDP (3 mg·kg^− 1^·d^− 1^) group; (6) CDDP (3 mg·kg^− 1^·d^− 1^) plus luteolin low-dose (10 mg·kg^− 1^·d^− 1^) group; (7) CDDP (3 mg·kg^− 1^·d^− 1^) plus luteolin medial-dose (20 mg·kg^− 1^·d^− 1^) group; (8) CDDP (3 mg·kg^− 1^·d^− 1^) plus luteolin high-dose (40 mg·kg^− 1^·d^− 1^) group. The CDDP were intraperitoneal injected once daily, and luteolin were given by gavage once daily for 5 days. The tumor volume was measured three times a week. Three weeks after treatment, the mice were sacrificed, and the tumor volume and weight were measured. The tumor tissues were used for histopathologic examination.

### TUNEL

Tumor paraffin tissue sections were processed with TUNEL assay to analyze apoptosis. The procedure was performed according to instructions of the TUNEL kit (KeyGen, Nanjing, China). The samples were observed under a microscope at × 100 magnification. The apoptotic cells were counted in three random fields for each sample, and the apoptosis percentage was calculated as follows: (Number of TUNEL-positive cells/Total number of cells in the field) × 100%.

### Drug combination effect analysis

Combination effect between the luteolin and cisplatin was analyzed by the Zheng-Jun Jin method [19–21]. In this method, the combination rate was evaluated by the inhibition rate via the Q value. The formula for the Q value is: Q = Ea + b / (Ea + Eb - Ea × Eb), where Ea + b, Ea, and Eb are the inhibition rate of the combination group, drug a and drug b, respectively. Q = 1 would mean simple addition; Q > l, synergism or potentiation, Q < 1, antagonism.

### Statistical analysis

All the experiments were repeated three times. The data were presented as mean ± SD. The difference between indicated groups were analyzed using Student’s t-test. *P* < 0.05 was considered be statistically significant.

## Results

### Luteolin dose dependently enhanced the proliferation inhibition effect of cisplatin in CAOV3/DDP cells

**Fig. 1:**
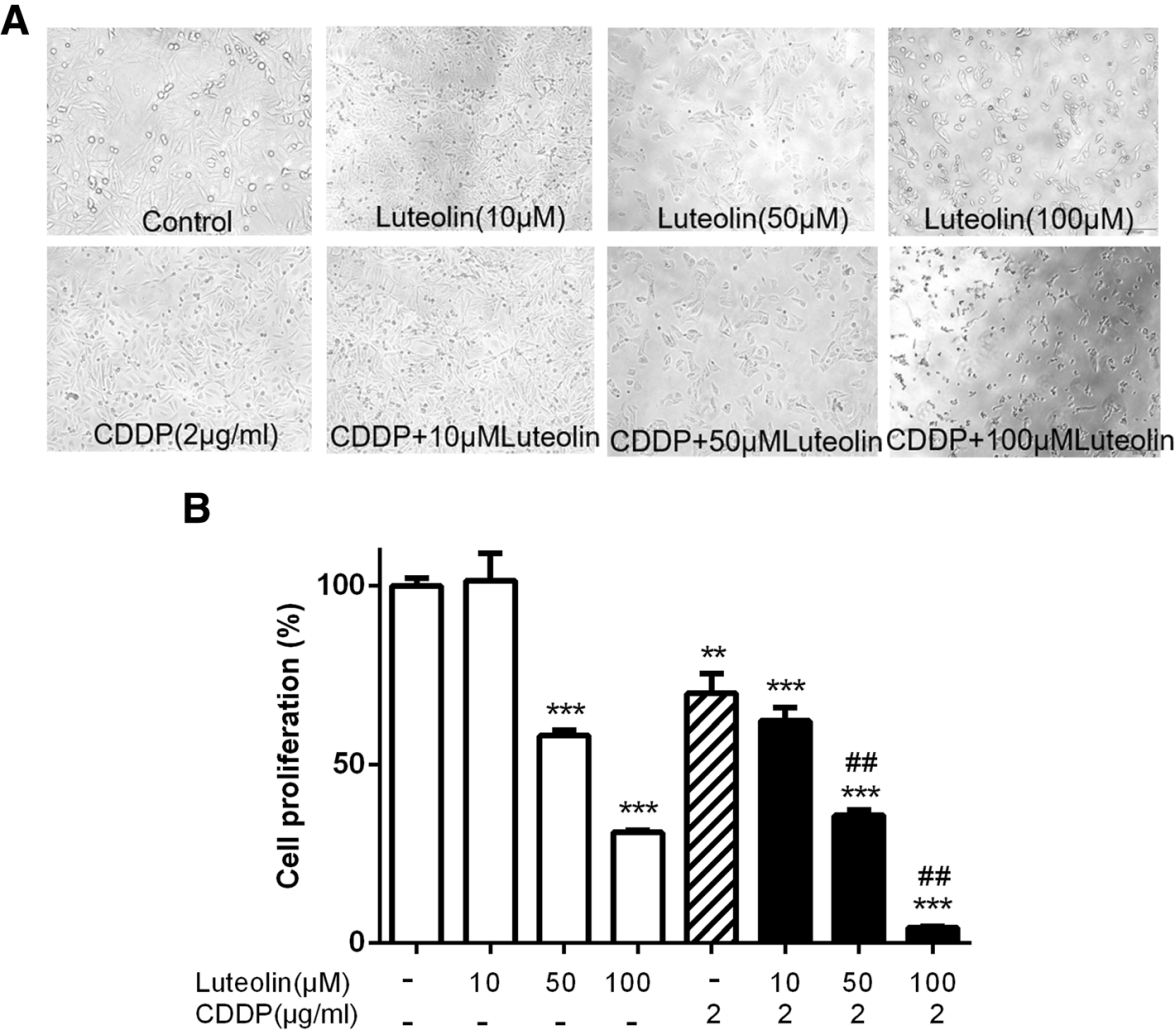
Effects of luteolin and cisplatin on the proliferation of CAOV3/DDP cells. Cells were treated with indicated concentrations of luteolin or cisplatin or both for 48 h, and cell proliferation was measured by CCK-8 assay. **a** Representative morphological changes of indicated treatment at × 200 magnification; **b** Dose response curves indicated significant reduction of cell proliferation in comparison to normal control. Data were represented as mean ± standard error of three independent experiments. ** *P* < 0.01, *** *P* < 0.001, vs. control; ## *P* < 0.01 vs. cisplatin. CDDP: cisplatin

Cells were treated with various doses of luteolin (0, 10, 50, and 100 μM), cisplatin (2 μg/ml) alone or in combination for 48 h and then cell proliferation was monitored by CCK-8 assay. As shown in Fig 1a, b, luteolin alone inhibited the cell proliferation of CAOV3/DDP cells in a concentration- dependent manner. Cells treated with combination of cisplatin (2 μg/ml) and luteolin (10, 50, 100 μM) for 48 h showed a more significant proliferation decrease in contrast with either luteolin or cisplatin alone. These results suggested that luteolin enhanced the proliferation inhibition effect of cisplatin in CAOV3/DDP cells in a concentration-dependent manner. To further investigate the nature of the combination effect between luteolin and cisplatin on CAOV3/DDP cells, the Q value was calculated based on the CCK-8 assay. As shown in Table 2, the data suggested that luteolin exhibited an additive or synergistic effect when combined with cisplatin.

**Table 2:** Luteolin increased the sensitivity of CAOV3/DDP cells to cisplatin. The Q value was calculated to evaluate the effect of the combination of the two drugs. The inhibition rates were measured by CCK-8 assay. CDDP combined with luteolin (100 μM) showed a synergistic inhibitory effect on the proliferation of CAOV3/DDP cells (Q = 1.22 ± 0.04, > 1, P < 0.01). The data were expressed as the mean ± S.D. in triplicate

Drugs	Inhibition rate (%)	Q value
Luteolin (100 μM)	69.1 ± 0.55	
Luteolin (50 μM)	42.0 ± 1.20	
Luteolin (10 μM)	−1.5 ± 6.26	
CDDP (2 μg/ml)	30.2 ± 4.54	
Luteolin (100 μM) + CDDP (2 μg/ml)	95.7 ± 0.24	1.22 ± 0.04
Luteolin (50 μM) + CDDP (2 μg/ml)	64.3 ± 1.22	1.08 ± 0.06
Luteolin (10 μM) + CDDP (2 μg/ml)	37.9 ± 3.02	1.36 ± 0.41

### Luteolin enhanced cisplatin induced apoptosis in CAOV3/DDP cells

**Fig. 2:**
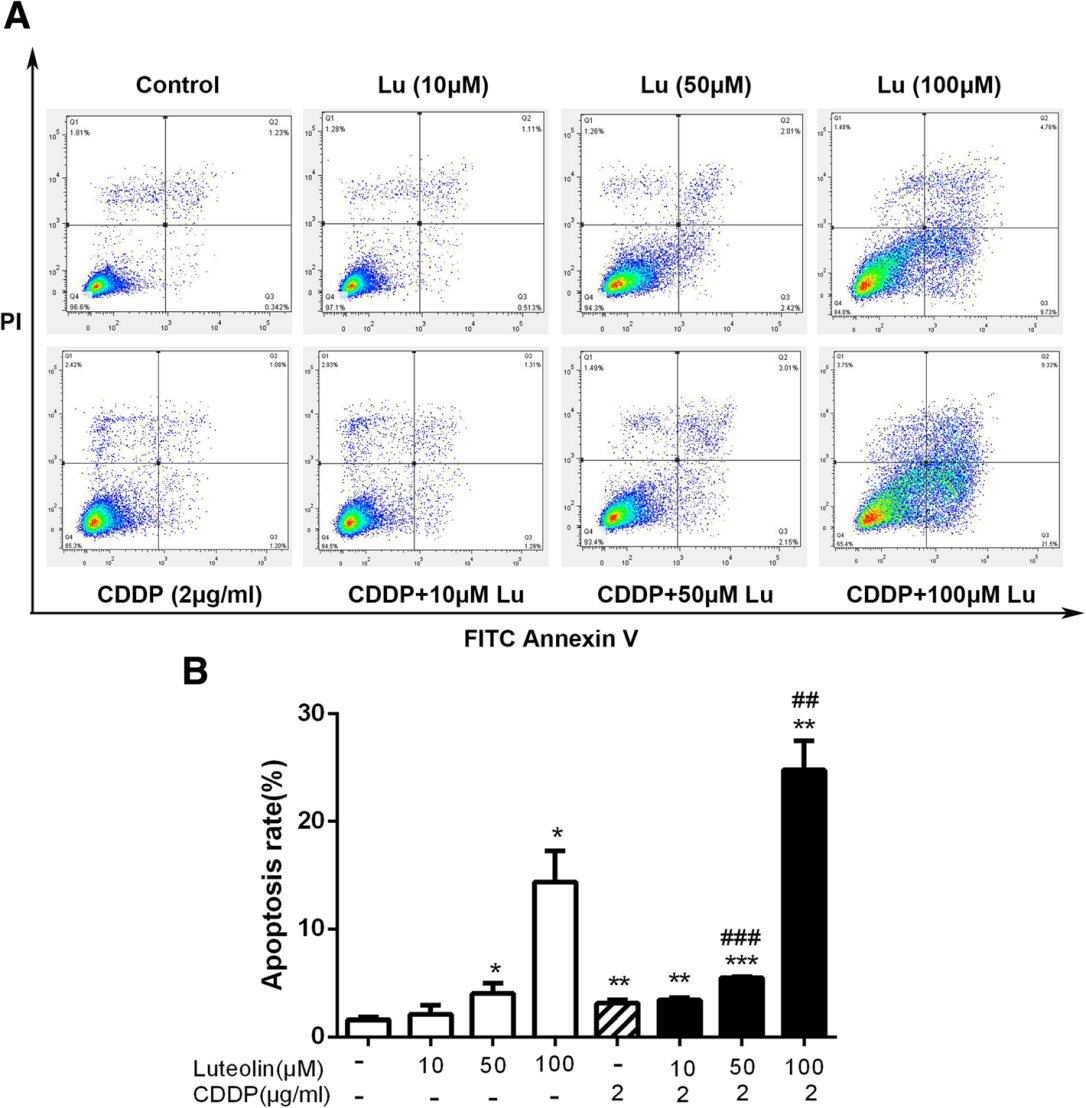
Luteolin induced cell apoptosis and enhanced cisplatin-induced apoptosis of CAOV3/DDP cells. Cells were treated with luteolin or cisplatin or in combination for 48 h, and then the apoptosis was detected by Annexin V/PI. **a** Flowcytometric analysis; **b** Statistical analysis for apoptosis ratio in each group. Data were represented as mean ± SD of three independent experiments. * *P* < 0.05, ** *P* < 0.01, *** *P* < 0.001, vs. control; ## *P* < 0.01, ### *P* < 0.001 vs. cisplatin. CDDP: cisplatin. CDDP combined with luteolin (100 μM) showed a synergistic effect on the apoptosis induction of CAOV3/DDP cells (Q = 1.46 ± 0.1, > 1, *P* < 0.01)

As luteolin promoted cisplatin induced cell proliferation inhibition, we further determined whether the combination treatment could exert synergic induction on cell apoptosis. Cell apoptosis was evaluated by flow cytometry following treatment of luteolin (0, 10, 50, and 100 μM), CDDP (2 μg/ml) alone or the combined treatments. As shown in Fig. 2a-b, no significant apoptosis was observed in cells treated with 10 μM luteolin. Treatments with higher doses (50 μM and 100 μM) of luteolin induced evident cell apoptosis, and the apoptosis rates were 4.29% and 14.39% respectively. Cisplatin alone caused about 3.11% of apoptosis. When cells were treated with both luteolin and cisplatin, the apoptosis rate increased significantly. The apoptosis rates of luteolin (10 μM) + cisplatin, luteolin (50 μM) + cisplatin and luteolin (100 μM) + cisplatin group were 3.41%, 5.48% and 24.75%, respectively.

### Luteolin and cisplatin decreased Bcl-2 expression synergistically

**Fig. 3:**
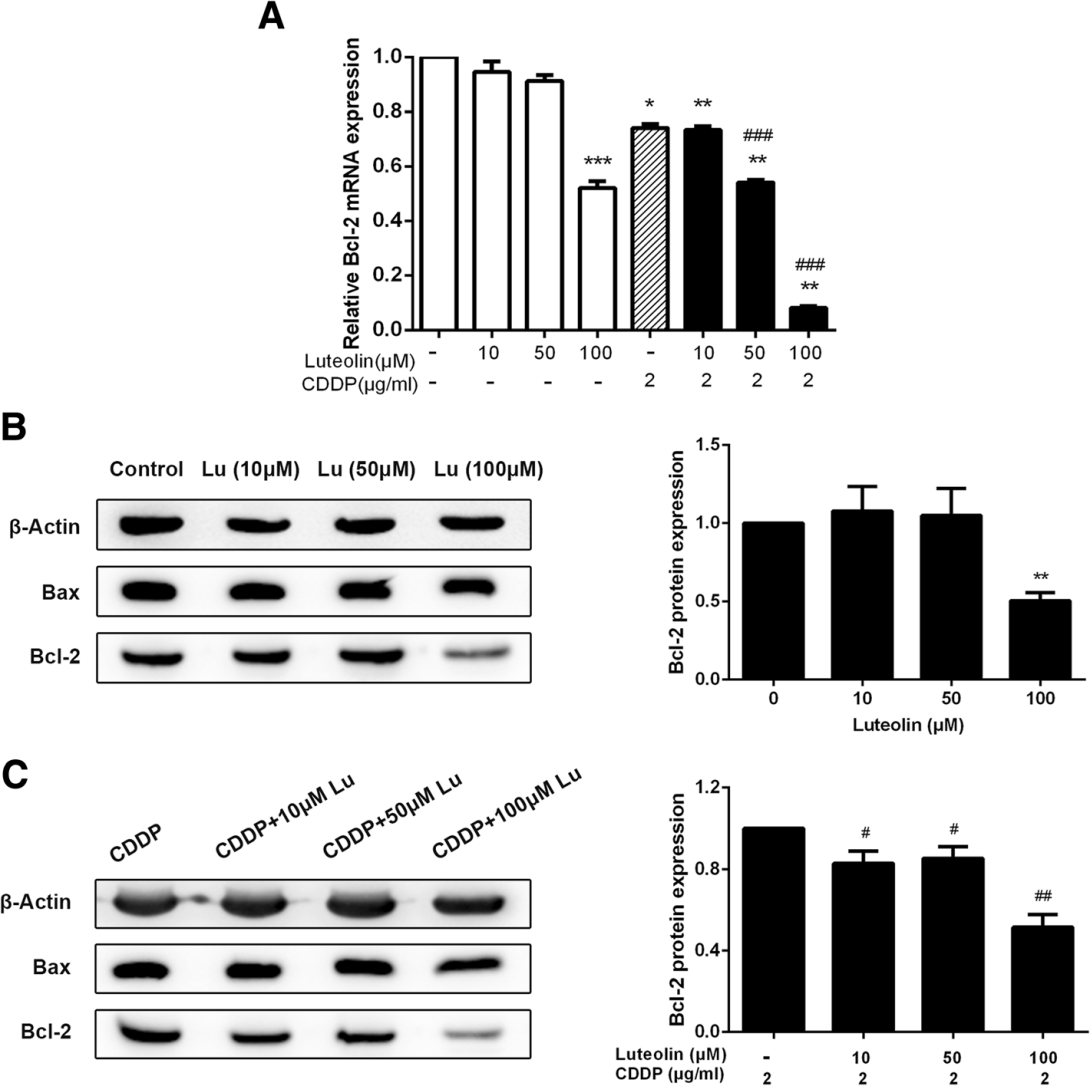
Effects of luteolin in combination with cisplatin on expression of apoptosis related proteins. CAOV3/DDP cells were treated with various concentrations of luteolin or cisplatin or the combination of both for 48 h, and then the expression of Bax, Bcl-2 was assessed by qRT-PCR and western blotting. **a** Relative Bcl-2 mRNA expression was normalized to β-actin; **b** Bax and Bcl-2 protein expressions of cells treated with luteolin; **c** Bax and Bcl-2 protein expressions of cells treated with the combination of cisplatin and increasing doses of luteolin. * *P* < 0.05, ** *P* < 0.01, *** *P* < 0.001, vs. control; # *P* < 0.05, ## *P* < 0.01, ### *P* < 0.001 vs. cisplatin. CDDP: cisplatin. CDDP combined with luteolin (50, 100 μM) indicated a synergistic inhibitory effect on the Bcl-2 expression of CAOV3/DDP cells (Q = 1.43 ± 0.16 and 1.50 ± 0.09, respectively, > 1, *P* < 0.01 and P < 0.001, respectively)

Next, to explore the underlying mechanisms involved in the sensitization effect of luteolin on cisplatin-induced apoptosis, we measured the expression level of the anti-apoptotic regulator, Bcl-2, by qRT-PCR and western blotting, and the pro-apoptotic protein Bax through western blotting assay. As shown in Fig. 3, luteolin at high dose of 100 μM decreased the Bcl-2 mRNA level and protein expression, and cisplatin alone also decreased the Bcl-2 level. Moreover, the Bcl-2 expression was decreased further in the combined treatment of luteolin and cisplatin. However, the Bax protein expression didn’t show significant change in all the groups (data not shown). These results suggested that luteolin enhanced the antitumor response of cisplatin by modulating apoptosis pathway.

### Luteolin combined with CDDP inhibited migration and invasion in CAOV3/DDP cells

**Fig. 4:**
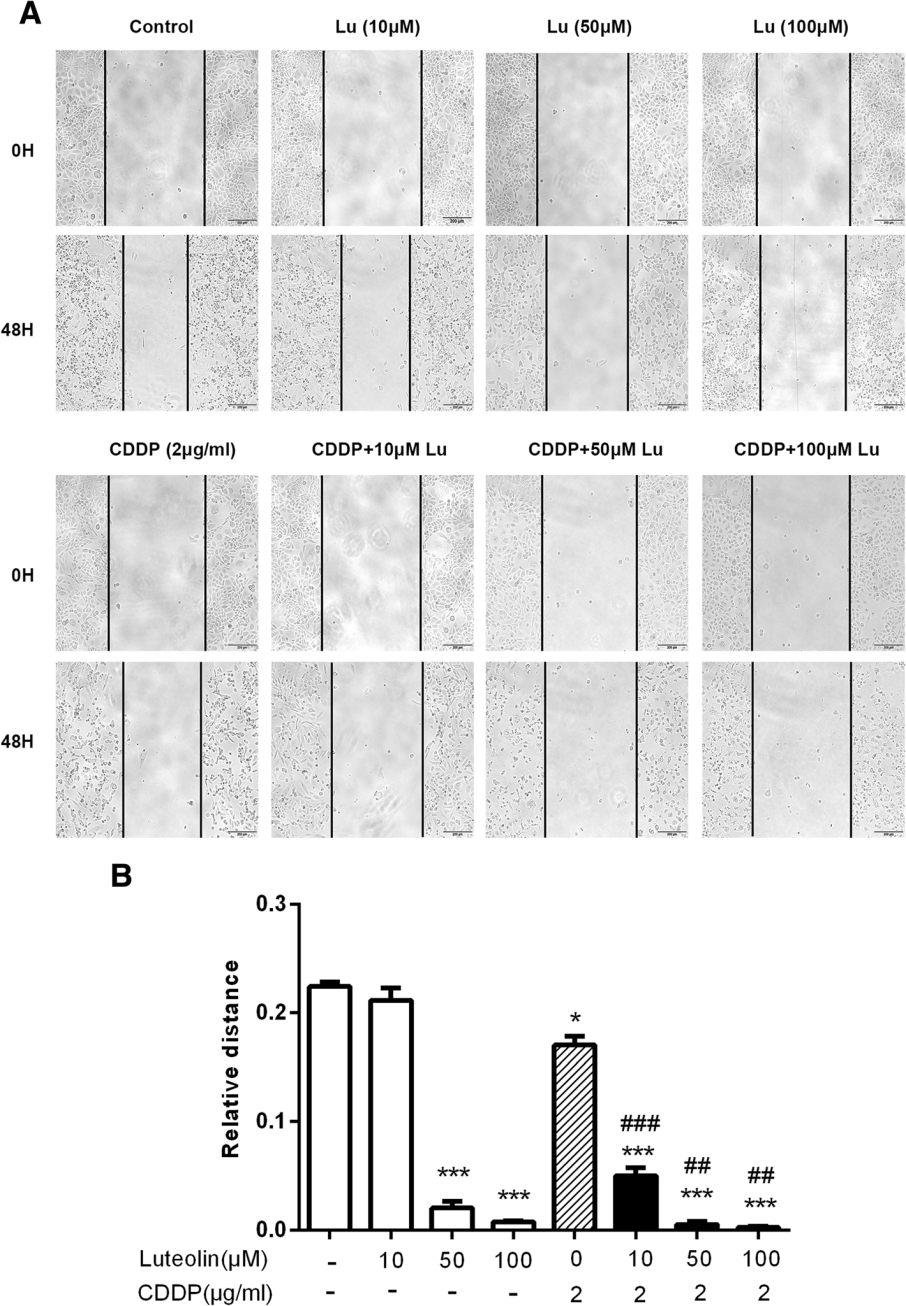
Luteolin inhibited cell migration and enhanced cisplatin-induced migration suppression in CAOV3/DDP cells. Migratory ability of CAOV3/DDP cells treated with increasing doses of luteolin or cisplatin or the combination of both agents was tested using wound-healing assay. **a** The gap of indicated groups was imaged at 0 and 48 h (magnification, × 100); **b** Relative migration distance of three independent experiments. * *P* < 0.05, *** *P* < 0.001, vs. control; ## *P* < 0.01, ### *P* < 0.001 vs. cisplatin. CDDP: cisplatin. CDDP combined with luteolin (10, 100 μM) showed a synergistic inhibitory effect on the migratory ability of CAOV3/DDP cells (Q = 2.91 ± 0.97 and 1.02 ± 0.003, respectively, > 1, *P* < 0.05 and *P* < 0.01, respectively)

**Fig. 5:**
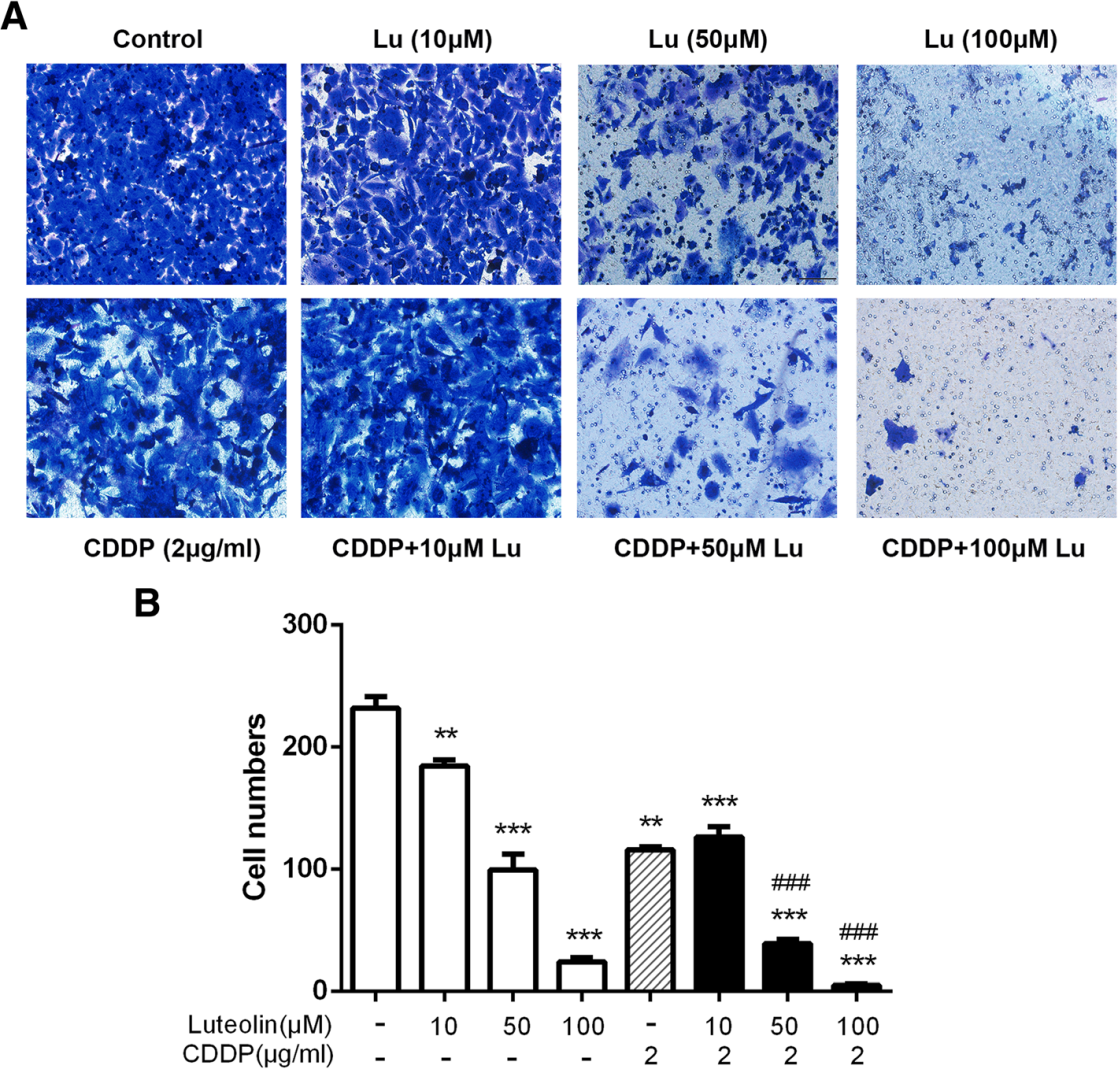
Luteolin suppressed cell invasion and enhanced cisplatin-induced suppression of invasion in CAOV3/DDP cells. Invasion ability of CAOV3/DDP cells of indicated treatments was measured using Matrigel invasion assay. **a** The image of invaded cells (magnification,× 200); **b** Numbers of invaded cells in each group of three independent experiments. ** *P* < 0.01, *** *P* < 0.001, vs. control; ### *P* < 0.001 vs. cisplatin. CDDP: cisplatin. CDDP combined with luteolin (50, 100 μM) showed a synergistic inhibitory effect on the invasion of CAOV3/DDP cells (Q = 1.06 ± 0.02 and 1.03 ± 0.007, respectively, > 1, *P* < 0.05 and *P* < 0.01, respectively)

To determine whether combination treatment affected cell migration and invasion ability, we then treated CAOV3/DDP cells with luteolin or cisplatin or combination of both by wound-healing assay and Matrigel invasion assay. The results (Figs. 4 and 5) showed that, luteolin alone inhibited cell migration and invasion in a dose-dependent manner, and the combination of CDDP and luteolin evidently decreased cell migration and invasion compared with either single agent treatment. These results demonstrated that luteolin could suppress migration and invasion and enhance sensitivity to CDDP in CAOV3/DDP cell line.

### Luteolin enhanced the anticancer effect of CDDP on ovarian cancer in vivo

**Fig. 6:**
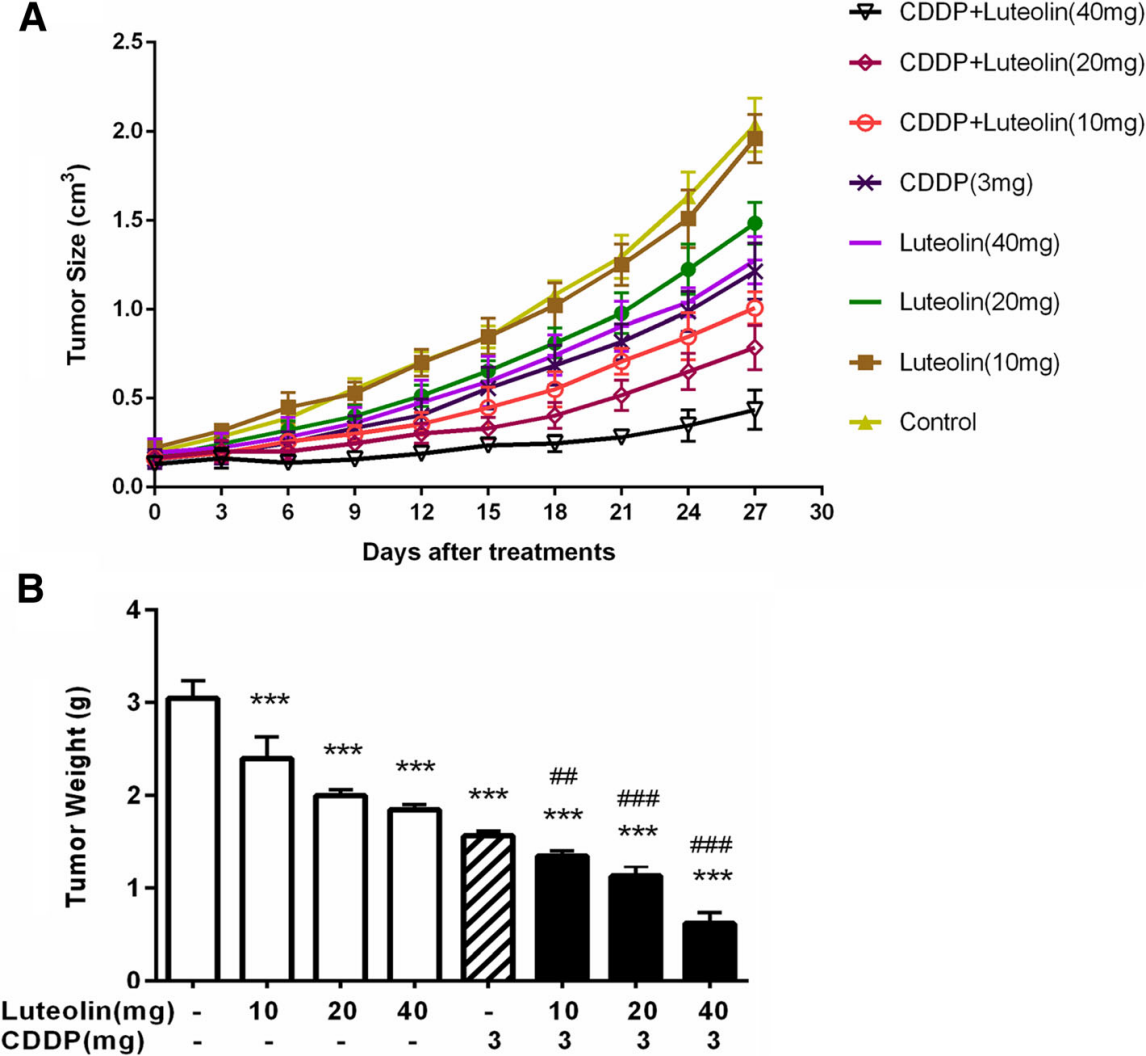
Luteolin enhanced antitumor efficacy of CDDP against xenograft model of ovarian cancer. Xenograft mice were treated with various doses of luteolin or cisplatin or in combination. **a** The tumor volume was measured three times a week. (*n* = 6). **b** Three weeks after treatment, the mice were sacrificed, and tumor weight were measured. (n = 6). *** *P* < 0.001, vs. control; ## *P* < 0.01, ### *P* < 0.001 vs. cisplatin. CDDP: cisplatin

To determine whether luteolin could enhance the cytotoxicity of CDDP in vivo, we established an ovarian cancer model in nude mice and investigated the therapeutic effects of luteolin alone or in combination with CDDP. The results showed that luteolin combined with CDDP notably impeded the tumor growth compared with cisplatin alone, exhibited as decreased tumor volume (Fig. 6a) and declined tumor weight (Fig. 6b). According to the tumor weight, we calculated the inhibition rate of each group, the combination tumor growth inhibition rate also showed a synergistic or additive effect (Table 3). These results were in consistent with in vitro experiments. Collectively, these results indicated that luteolin enhanced CDDP sensitivity of ovarian cancer in vivo.

**Table 3:** Luteolin increased the sensitivity of xenograft model of ovarian cancer to cisplatin. The Q value was calculated to evaluate the effect of the combination of the two drugs. The inhibition rate in each group was measured by tumor weight reduction compared to the control group. CDDP combined with luteolin (40 mg) showed a synergistic inhibitory effect on the growth of xenograft tumor (Q = 1.16 ± 0.03, > 1, *P* < 0.01). The data were expressed as the mean ± S.D. in triplicate

Drugs	Inhibition rate (%)	Q value
Luteolin (40 mg)	39 ± 1.64	
Luteolin (20 mg)	34.4 ± 1.89	
Luteolin (10 mg)	21.3 ± 6.83	
CDDP (3 mg)	48.6 ± 1.55	
Luteolin (40 mg) + CDDP (3 mg)	79.8 ± 3.5	1.16 ± 0.03
Luteolin (20 mg) + CDDP (3 mg)	62.8 ± 3.09	0.95 ± 0.03
Luteolin (10 mg) + CDDP (3 mg)	55.7 ± 1.64	0.94 ± 0.05

### Combined treatment of CDDP with luteolin increases xenograft tumor cell apoptosis

**Fig. 7:**
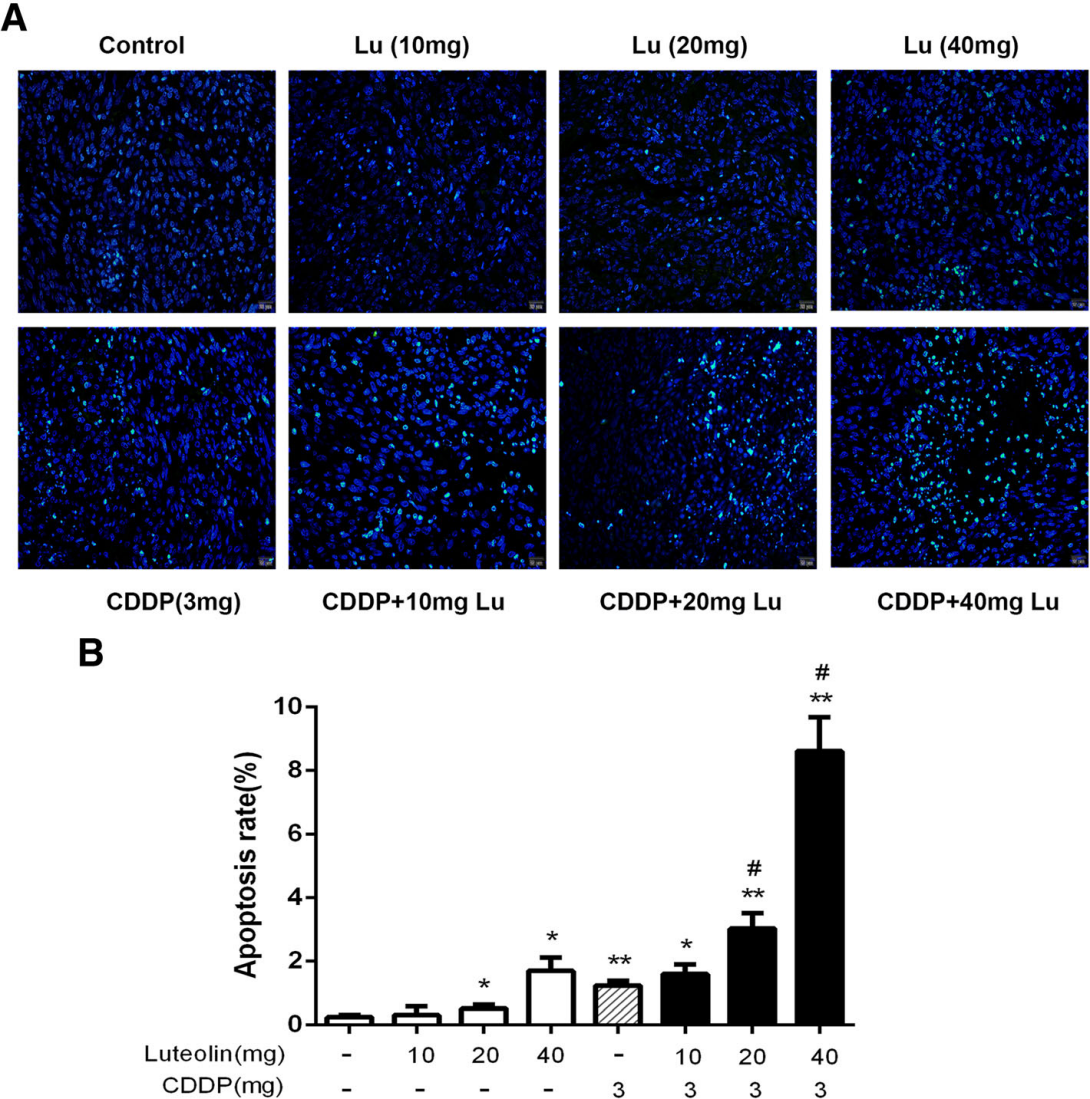
Luteolin in combination with cisplatin enhanced apoptosis in vivo. Apoptosis of tumor sections were detected by TUNEL assay. **a** Representative images of apoptotic cells in each group (apoptotic cells in green and the cell nuclei in blue). **b** The tumor cell apoptosis rates of 8 groups were analyzed. * *P* < 0.05, ** *P* < 0.01, vs. control; # *P* < 0.05 vs. cisplatin. CDDP: cisplatin. CDDP combined with luteolin (20 mg, 40 mg) exhibited a synergistic effect on the apoptosis induction of xenograft tumor (Q = 1.73 ± 0.03 and 2.95 ± 0.16, respectively, > 1, *P* < 0.01 and *P* < 0.01, respectively)

Further, we examined the effect of combined treatment of CDDP with luteolin on tumor cell apoptosis through TUNEL assay in the tumor tissues isolated from the 8 groups of mice above. As shown in Fig. 7, luteolin alone induced apoptosis at doses of 20 mg·kg^− 1^·d^− 1^ and 40 mg·kg^− 1^·d^− 1^ (the apoptosis rates were 0.51% and 1.70%, respectively) while the lower dose at 10 mg·kg^− 1^·d^− 1^ didn’t show significant effect compared with control group (apoptosis rate: 0.24%). The results also revealed an increased apoptosis rate by combined treatment compared with cisplatin treatment alone. The apoptosis rates of CDDP, CDDP plus low dose of luteolin, CDDP plus medial dose of luteolin and CDDP plus high dose of luteolin were 1.24%, 1.59%, 3.03%, and 8.61%, respectively. This further demonstrated that luteolin enhanced antitumor effect of CDDP by increasing apoptosis of tumor cells.

## Discussion

Cisplatin is one of the most effective therapeutic agents widely used in clinic for the treatment of EOC. However, drug resistance is a major problem that limits its clinical application. Therefore, combination treatment with new sensitizing agents is an effective strategy to overcome cisplatin resistance [10]. Luteolin, a flavonoid that has been identified in many plants, has demonstrated in numbers studies to exhibit chemopreventive or chemosensitising properties against various human cancers. In the current study, we provide experimental evidence both in vivo and in vitro that luteolin is able to enhance the therapeutic potential of cisplatin in ovarian cancer.

In the current study, firstly, we evaluated the effect of luteolin or cisplatin or the combination of both on the cell proliferation in human cisplatin-resistant ovarian cancer CAOV3/DDP cells. We found that luteolin alone inhibited the cell proliferation in a dose-dependent manner, and co-treatment with both agents could further decrease cell proliferation. These results suggested that luteolin could exert synergistic anti-proliferation effect with cisplatin in CAOV3/DDP cells.

Apoptosis inhibition is one of the main mechanisms responsible for the resistance of chemotherapy [22]. Cisplatin is one of the most effective drugs for the treatment of ovarian cancer, and the mechanism involved in the process of its cytotoxicity include survival inhibition and apoptosis induction. Once the apoptotic pathway is blocked, tumor cells acquire resistance to pro-apoptotic effect of cisplatin, which reduces the antitumor effect of cisplatin [23]. Therefore, inhibition of apoptosis is an effective strategy to overcome the drug resistance and promote the anti-tumor effect of cisplatin [24]. Luteolin has been reported to induce apoptosis in various cancer cells such as human cervical cancer cells [13], esophageal carcinoma cells [25] and colorectal cancer cells [26]. Our study found that the single treatment with luteolin could dose-dependently induce apoptosis in CAOV3/DDP cells, when combined with cisplatin, luteolin could significantly enhance cisplatin-induced cell apoptosis, indicating that luteolin enhanced the sensitivity of cisplatin, in part, through apoptosis induction.

The BCL-2 protein family plays a key role in the regulation of cell apoptosis. The BCL-2 protein family can be divided into three different subfamilies, including pro-survival factions such as BCL-2, MCL1 and BCL-XL, which inhibit the apoptosis process, and two pro-apoptotic subfamilies, the death effectors BAX and BAK and the BH3-only proteins such as BID, BIM and PUMA, which contribute to cell apoptosis [27–29]. Consequently, the ratio of Bcl-2/Bax is an essential factor to determine whether a tumor cell commits apoptosis or not. Overexpression of Bcl-2 can inhibit cell apoptosis, lead to resistance to cisplatin, and result in poor prognosis of cancer patients. Recent study has demonstrated that Bcl-2 is overexpressed in ovarian cancer [30, 31] and has a significant positive correlation with sensitivity to cisplatin in ovarian cancer cells [32]. Therefore, targeting Bcl-2 may provide an effective therapeutic method to solve drug resistance in ovarian cancer. It was previously reported that luteolin could decrease Bcl-2 expression in various cancer cells [33]. In the current study, results from qRT-PCR showed that luteolin at high concentration (100 μM) could significantly decrease the Bcl-2 mRNA level, and the combination of luteolin with cisplatin could evidently inhibit Bcl-2 expression compared with cisplatin alone. This suggests that the combined treatment induced cell apoptosis through the inhibition of Bcl-2 expression. The BCL-2 family proteins control the permeability of mitochondria and the release of cytochrome c to the cytoplasm, following the activation of a group of caspases, which proceeds apoptosis [27]. This suggests that mitochondrial apoptosis pathway may be involved, and further study should be focused on the pathway.

Our data also revealed the potent antitumor effect of luteolin with cisplatin in ovarian cancer in vivo. Single treatment with increasing doses of luteolin showed growth inhibition in xenograft tumor. In addition, tumor volume and weight were significantly decreased in mice of combination treatment group compared with cisplatin alone. What’s more, the combination therapy synergistically induced more apoptosis than cisplatin, which is in consistent with in vitro study. These results further demonstrate that the inhibition of tumor growth was induced, in part, by the enhancement of cisplatin induced apoptosis.

Ovarian cancer is highly susceptible to occur metastasis in late stage. In most patients, though appearance of the lesion is still localized in the ovary, subclinical metastasis may already exist in many parts of the peritoneal or omentum [34]. In addition, chemotherapy resistance leads to the decrease of chemotherapy sensitivity in ovarian cancer cells, and also enhance its malignant degree. It suggests that the occurrence of chemotherapy resistance is closely related to the promotion of invasion and metastasis in cancer cells [35, 36]. Cancer metastasis involves several processes including adhesion, migration, and invasion. Targeting these processes provides effective strategy to enhance the chemosensitivity of cisplatin [37]. Luteolin has been proven to inhibit metastasis in various caner types such as breast cancer [38] and prostate cancer [39]. In our experiment, wound-healing assay and Matrigel invasion assay showed that luteolin exhibited a dose-dependent suppression on migration as well as invasion in CAOV3/DDP cells. Additionally, the inhibition effect became stronger when treated the cells with both increasing concentrations of luteolin and cisplatin than single agent treatment. These results indicate that the improved anticancer effect of cisplatin in CAOV3/DDP cells by luteolin is partially mediated through inhibition in cell migration and invasion.

In conclusion, our study shows that luteolin, a natural flavonoid, significantly enhances the anti-tumor effect of cisplatin in ovarian cancer both in vivo and in vitro. Combination of luteolin and cisplatin is more effective in suppressing CAOV3/DDP cell growth and metastasis. Luteolin could enhance cisplatin induced apoptosis in cisplatin-resistant ovarian cancer CAOV3/DDP cells via decreasing Bcl-2 expression. Our preliminary data provide experimental evidence for potential clinical application of luteolin as a novel chemosensitizer in the chemotherapy in ovarian cancer.
